# The Influence of Topiramate on Morphine Dependence in Mice

**DOI:** 10.3390/biom15050730

**Published:** 2025-05-16

**Authors:** Adrian Pysiewicz, Antonina Mazur, Jolanta Kotlińska, Irena Baranowska-Bosiacka, Krzysztof Fronc, Małgorzata Łupina, Marta Kruk-Słomka, Joanna Listos

**Affiliations:** 1 Department of Pharmacology and Pharmacodynamics, Medical University of Lublin, Chodźki 4a St., 20-093 Lublin, Poland; krzysztof.michal.fronc@gmail.com (K.F.); marta.kruk-slomka@umlub.pl (M.K.-S.); joanna.listos@umlub.pl (J.L.); 2 Department of Pharmaceutical Sciences, Jan Kochanowski University, al. IX Wieków Kielc 19a, 25-516 Kielce, Poland; antonina.mazur@ujk.edu.pl; 3 Department of Biochemistry and Medical Chemistry, Pomeranian Medical University, Powstańców Wlkp. 72 Av., 70-111 Szczecin, Poland; irena.baranowska.bosiacka@pum.edu.pl; 4 Department of Experimental and Clinical Pharmacology, Medical University of Lublin, Jaczewskiego 8B, 20-090 Lublin, Poland; malgorzata.lupina@umlub.pl

**Keywords:** topiramate, morphine withdrawal signs, naloxone, morphine tolerance, hot-plate test, behavioral sensitization

## Abstract

Topiramate evokes pharmacological activity via a blockade of voltage-dependent sodium channels, reduction in glutamate release, inhibition of AMPA receptors and kainate receptors, and potentiation of GABAergic neurotransmission. Therefore, it is used not only as an antiseizure drug but is also effective in migraine prophylaxis, cluster headaches, neuropathic pain, and alcohol dependence. The aim of this study was to investigate the effect of topiramate in morphine dependence in mice, particularly in terms of morphine tolerance, morphine withdrawal signs, and morphine sensitization. In these experiments, topiramate was administered both acutely and chronically. Topiramate significantly reduced the morphine tolerance in the hot-plate test and attenuated naloxone-induced morphine withdrawal signs. Its effect on morphine sensitization to the locomotor activity of mice was poor. The obtained results showed that topiramate might be an effective drug for reducing the physical symptoms of morphine dependence.

## 1. Introduction

Topiramate is a second-generation antiseizure drug. It is used in the therapy of epilepsy, mainly in tonic-clonic seizure incidents and in secondary generalized seizures. Topiramate is not used in status epilepticus because its pharmacological action is delayed [1,2]. Topiramate belongs to the class of sulfamate-substituted monosaccharides derived from D-fructose and is structurally unrelated to other antiepileptic drugs. Its diversified pharmacological activity is attributed to multifaceted mechanisms of action. As the major topiramate target is considered to be the blockade of voltage-dependent sodium channels, it results in the inhibition of Na+ influx and in the shortening of the duration and frequency of action potentials [3,4,5]. This effect is reversible and stronger in comparison with carbamazepine, a classic antiepileptic drug [6]. Topiramate also has the ability to inhibit receptors for excitatory amino acids, i.e., AMPA receptors and kainate receptors [7,8,9,10]. Its effect on NMDA receptors seems to be weaker. Topiramate also reduced glutamate levels in the hippocampus of epileptic rats [11,12]. Moreover, it was confirmed that GABAergic transmission was also involved in topiramate’s activity. It was able to increase endogenous GABA levels [13] and to increase GABAA receptor activity via the β2, β3, and α subunits [14,15]. Topiramate also increased the frequency but not the duration of chloride channel opening [16,17,18].

Taking into account all topiramate mechanisms, the possibilities of clinical topiramate application are also steadily extended. It was evidenced that topiramate was effective in migraine prophylaxis (both in migraines with and without auras) [19,20], as it reduced the frequency and intensity of migraine attacks and improved the overall quality of life of patients [21,22,23]. Therefore, in many countries, topiramate has been approved for migraine prophylaxis in adults. Topiramate-induced analgesia was also observed in cluster headaches [20], in diabetic neuropathic pain [24], in trigeminal neuralgia [25], and in patients with multiple sclerosis [26]. After chronic topiramate administration, a body weight loss was noticed. Currently, in some countries, including the United States, a combination of topiramate and phentermine (an adrenergic agonist) has been registered for the therapy of overweight and obesity in people, as a supplement to a low-energy diet and physical activity [27,28,29].

The topiramate mechanism involving the activation of GABAergic system and inhibition of glutamatergic system may stabilize neurons in brain and contribute to the reduction in dopamine release in the mesocorticolimbic system. It suggests that topiramate may produce a beneficial effect in drug addiction. Currently is known that topiramate is a promising drug in the treatment of alcohol dependence [30,31,32], and also to a lesser extent for cocaine dependence [33,34], as well as in behavioral addiction consisting of binge eating disorder [35,36]. The data on the significance of topiramate in morphine dependence are poor.

Morphine dependence is classified as a chronic recurrent disease in the central nervous system which produces serious consequences, including personality disorders, the development of comorbidities, and even premature death. The rewarding effect of morphine is associated with the stimulation of µ receptors, particularly those located on GABAergic terminals in the VTA, which results in the inhibition of endogenous GABA secretion. This leads to the disinhibition of dopaminergic neurons, increased dopamine secretion in the nucleus accumbens, and a feeling of euphoria that promotes the development of physical and psychological dependence [37].

At present, methadone or buprenorphine are usually used as a replacement therapy to treat morphine dependence. Another drug, naltrexone, is recommended for the suppression of morphine withdrawal symptoms. Additionally, other compounds which attenuate negative symptoms of morphine withdrawal can be used, such as clonidine (for anxiety), paracetamol (for pain), or loperamide (for diarrhea). It is worth noting that these drugs are far from ideal—it is estimated that their effectiveness is about 30%, and they produce adverse effects (for example clonidine-induced hypotension). Therefore, innovative opportunities are sought. Taking into account the pharmacology of topiramate and its diversified clinical applications, the consideration of this compound in the therapy of morphine dependence seems to be justified [38]. However, despite its many advantages, topiramate also has adverse effects. For instance, it has teratogenic activity. Moreover, it may produce sudden myopia and secondary angle-closure glaucoma. Symptoms of the syndrome include sudden decreased visual acuity and/or ocular pain. Therefore, in patients with a history of visual impairment, topiramate should be used with caution. In some cases, it may also induce metabolic acidosis, which increases the risk of kidney stone formation and may lead to osteopenia. Although the essential interactions between topiramate and morphine have not been studied, it may be supposed that this combination may increase the risk of side effects. Dizziness, drowsiness, confusion, or difficulty in concentration may develop because both drugs affect the central nervous system. Therefore, it is certain that the administration of the drugs together requires monitoring of the side effects, but this therapy is not contraindicated [39].

Therefore, the purpose of that study was to investigate whatever topiramate would be effective in the attenuation of morphine dependence in mice, specifically in terms of morphine tolerance, morphine withdrawal signs, and morphine sensitization. Morphine tolerance is defined as the necessity to take a higher dose of morphine in order to achieve the same pharmacological effect [40]. It evokes a risk of acute intoxication or even death due to respiratory depression. In this study, the morphine tolerance to the antinociceptive effect was assessed using the hot-plate test. Morphine withdrawal symptoms usually develop after abrupt cessation of long-term administration of the drug or after administration of the opioid receptor antagonist naloxone [41,42]. Animals then develop jumpiness, paw tremors, teeth chattering, wet dog shakes, and diarrhea. In a previous study, morphine withdrawal symptoms were induced by naloxone administration, and the severity of morphine withdrawal syndrome was assessed based on the number of jumps. The intermittent administration of morphine in animals leads to the development of behavioral sensitization, which is expressed as an intensification of its pharmacological activity after a break episode. In animals, behavioral sensitization reflects drug-seeking behavior. In humans it often leads to relapses to drug use [43]. Therefore, in experimental pharmacology, behavioral sensitization is an important parameter for assessing psychological addiction, and it is usually manifested in animals as an increase in locomotor activity after exposure to a challenge dose of morphine [44,45]. This experimental model was used in that study.

Moreover, two paradigms of topiramate administration were used, namely an acute application (expression model) and a chronic application (simultaneously with morphine, with 5–8 doses depending on experimental procedure).

These experiments significantly expand the current state of knowledge on the pharmacological properties of topiramate. In the future, they may contribute to the broader clinical use of the studied drug.

## 2. Materials and Methods

### 2.1. General Experimental Conditions

The study was conducted on healthy male *Swiss albino mice* weighing 20–25 g, 25–30 day old. The animals were maintained on a standard diet (Murigran, Motycz, Poland) and had ad libitum access to food and water. The ambient temperature was constant at 22 ± 1 °C. Behavioral experiments were carried out between 8:00 a.m. and 4:00 p.m. In the case of the model of naloxone-induced morphine withdrawal, morphine injections were applicated between 8:00 a.m. and 8:00 p.m. A natural day–night light cycle (spring) was maintained. Prior to the experiments, the animals were acclimatized to the environmental conditions for 7 days. Mice were used in experiments only once, and following the completion of the study, they were euthanized by placing them in a carbon dioxide atmosphere.

The substances were applied at a volume of 0.1 mL per 10 g body weight (0.01 mL/g). Control animals received the same volume of solvent. The substances were administered intraperitoneally (i.p.). The number of animals per group (N) was 8 to 10 individuals.

The experiments were conducted in accordance with the guidelines of the European Parliament and Council Directive of 22 September 2010 (2010/63/EU) and were approved by the Local Ethics Committee (No. 1/2010, No. 20/2019, No. 33/2019).

### 2.2. Drugs

The following substances were used in the experiments: topiramate (Topamax, tablets, Janssen-Cilag International, Belgium), morphine hydrochloride (Pharma Cosmetic, Cracow, Poland), and naloxone hydrochloride (Sigma-Aldrich, Saint Louis, MO, USA). Topiramate was suspended in a 0.5% solution of methylcellulose while morphine hydrochloride and naloxone hydrochloride were dissolved in a 0.9% NaCl solution (B. Braun Melsungen, Melsungen, Germany). There were two control animal groups. The first received the 0.9% NaCl solution, while the second received the 0.5% methylcellulose solution. There were no significant changes in behavior between these groups. Therefore, the figures show only the results for the 0.5% methylcellulose solution (control group). All substances were intraperitoneally (i.p.) administered.

### 2.3. The Procedures of Behavioral Experiments

#### 2.3.1. The Effect of Topiramate (12.5; 25 mg/kg, i.p.) on Morphine Tolerance to the Antinociceptive Effects in the Hot-Plate Test in Mice

**Scheme 1:**
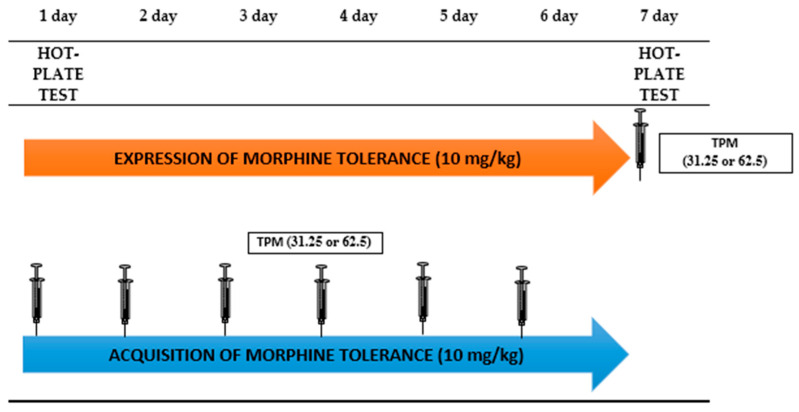
Graphical Presentation of Experimental Procedure of Topiramate Effect (12.5; 25 mg/kg, i.p.) on Morphine Tolerance to the Antinociceptive Effects in the Hot-Plate Test in Mice.

Tolerance to the antinociceptive effects of morphine was evaluated using the hot-plate test, based on procedure described by Fish et al. [46]. The apparatus consisted of a metal platform heated to a temperature of 55 ± 0.5 °C, surrounded by a transparent plexiglass cylinder with a diameter of 20 cm and a height of 18 cm. The experiment was conducted in a well-lit, soundproof room. Each mouse was placed individually on the hot platform, and the time (in seconds) to the first animal reaction (such as paw licking or jumps) was recorded. The maximal time of observation was 60 s, Scheme 1.

The morphine tolerance to its antinociceptive effect in mice was twice daily for seven consecutive days. The control group received a vehicle injection. The hot-plate test was conducted on days 1 and 7 of the experiment.

The impact of topiramate on the expression of morphine tolerance was assessed by the administration of an acute dose of topiramate on the 7th day of the experiment, 30 min prior to the morning morphine injection. Next, 30 min after the morphine injection, the hot-plate test was conducted.

The effect of topiramate on the acquisition of morphine tolerance was assessed by the administration of topiramate once a day for six consecutive days (from day 1 to day 6 of the experiment) in the morning, 30 min prior to the morning morphine dose. On day 7, mice received only a morphine injection, and 30 min later, the hot-plate test was conducted.

There were groups in this experiment: control (1st day), morphine (1st day), control (7th day), morphine (7th day), morphine+topiramate 12.5 (7th day), morphine+topiramate 25 (7th day), topiramate 12.5 (7th day), topiramate 25 (7th day).

#### 2.3.2. The Influence of Topiramate (12.5; 25 mg/kg, i.p.) on the Severity of Morphine Withdrawal Symptoms Induced by Naloxone (2 mg/kg, i.p.) in Mice

**Scheme 2:**
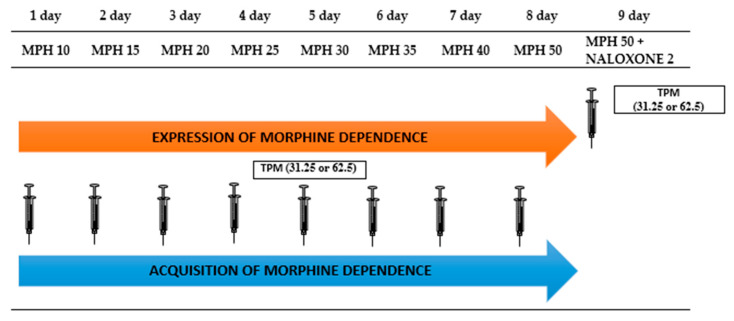
Graphical Presentation of Experimental Procedure of Topiramate Effect (12.5; 25 mg/kg, i.p.) on the Severity of Morphine Withdrawal Symptoms Induced by Naloxone (2 mg/kg, i.p.) in Mice.

The experimental procedure for the development of morphine dependence in mice was previously elaborated at the Department of Pharmacology and Pharmacodynamics [47,48]. Accordingly, morphine was administered at increasing doses (10, 15, 20, 25, 30, 35, 40, and 50 mg/kg, i.p.) twice daily (morning and evening) for eight consecutive days. On the ninth experimental day in the morning, an additional dose of morphine (50 mg/kg, i.p.) was administered. Then, one hour later, an opioid receptor antagonist, namely naloxone (2 mg/kg, i.p.), was injected to precipitate acute withdrawal syndrome. Immediately after naloxone administration, the mice were placed in 10 L glass cylinders for observation. The number of animal jumps was recorded over a 30 min period. The control group received a 0.5% methylcellulose solution (vehicle group), Scheme 2.

The effect of topiramate on the expression of morphine withdrawal symptoms was evaluated via the administration of single topiramate dose on the ninth day of the experiment, 10 min prior to morphine injection. Subsequently, 60 min after the morphine injection, naloxone was administered, and the number of jumps was recorded for a 30 min period.

The effect of topiramate on the acquisition of morphine withdrawal symptoms was assessed via the administration of topiramate once a day for eight consecutive days, 10 min prior to morphine injection. On the ninth day in the morning, the animals received a morphine injection, followed 60 min later by the administration of naloxone. Then, animal behavior (jumps) was observed for a 30 min period.

There were groups in this experiment: control, control+naloxone, morphine+naloxone, morphine+topiramate 12.5+naloxone, morphine+topiramate 25+naloxone, topiramate 12.5+naloxone, topiramate 25+naloxone.

#### 2.3.3. The Influence of Topiramate (12.5; 25 and 50 mg/kg, i.p.) on Morphine-Induced Sensitization to the Locomotor Effect in Mice

**Scheme 3:**
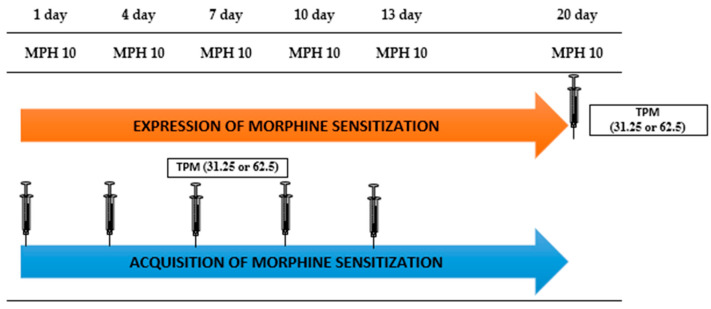
Graphical Presentation of Experimental Procedure of Topiramate Effect (12.5; 25 and 50 mg/kg, i.p.) on Morphine-Induced Sensitization to the Locomotor Effect in Mice.

The morphine sensitization to the locomotor effects in mice was assessed using the Kuribara method [49], modified by Kotlińska and Bocheński [50]. Sensitization was developed by the administration of five sporadic (every 72 h) morphine injections (10 mg/kg, i.p.) on days 1, 4, 7, 10, and 13 of the experiment. After each morphine injection, the animals were placed in actimeters to assess locomotor activity (60 min). On the 20th day of the experiment, 7 days after the morphine injection, the animals received a challenge dose of morphine (10 mg/kg, i.p.), and their locomotor activity was again measured over a 60 min period (test day). The control group received a 0.5% methylcellulose solution (vehicle group), Scheme 3.

The effect of topiramate on the expression of morphine sensitization in mice was investigated by the administration of topiramate on the 20th day of the experiment, 30 min prior to the morphine injections. Immediately after morphine administration, the animals were placed in actimeters for 60 min to measure locomotor activity. The influence of topiramate on the acquisition of morphine sensitization was studied via topiramate administration on days 1, 4, 7, 10, and 13 of the experiment (5 sporadic injections), 30 min prior to the morphine injections. On the 20th day of the experiment, the mice received only the morphine injection. After the drug application (on days 1, 4, 7, 10, 13 and 20), the animals were placed in actimeters for 60 min to measure locomotor activity. The control group received injections of a 0.5% methylcellulose solution (vehicle group).

There were groups in this experiment: control (1st day), morphine (1st day), control (20th day), morphine (20th day), morphine+topiramate 12.5 (20th day), morphine+topiramate 25 (20th day), morphine+topiramate 50 (20th day).

#### 2.3.4. Statistical Analysis

Statistical analysis was performed using GraphPad Prism Software (9.1.1). When our data followed a normal distribution, ANOVA analysis was performed. Two-way ANOVA was used to assess the morphine tolerance and morphine sensitization in mice. The morphine withdrawal was analyzed using one-way ANOVA. Comparisons between groups were made using Tukey’s test (post hoc). Results that probability ratio (*p*) was less than 0.05 (*p* < 0.05) were considered as statistically significant. In the figures results are presented as the mean ± SEM. The animals were randomly chosen to particular group. In the first step of the study the number of animals in each groups was 10 mice. For statistical analysis, if it was necessary (a result that deviates greatly from the average), maximal 2 results were removed (N = 8–10). Total number of animals used in experiments was 440 mice.

## 3. Results

### 3.1. The Effect of Topiramate (12.5; 25 mg/kg, i.p.) on Morphine Tolerance to the Antinociceptive Effects in the Hot-Plate Test in Mice

#### 3.1.1. The Influence of Topiramate on the Expression of Morphine Tolerance

Based on the two-way ANOVA, statistically significant changes in the reaction time to nociceptive stimulus were confirmed after acute and chronic morphine administration. A day effect (F(1,32) = 55.68; *p* < 0.0001), drug effect (F(1,32) = 97.13; *p* < 0.0001), and interaction effect (F(1,32) = 22.26; *p* < 0.0001) were observed. Additionally, one-way ANOVA showed statistically significant differences in animal behavior on the seventh day of the experiment (F(5,52) = 7.680; *p* < 0.0001).

**Figure 1:**
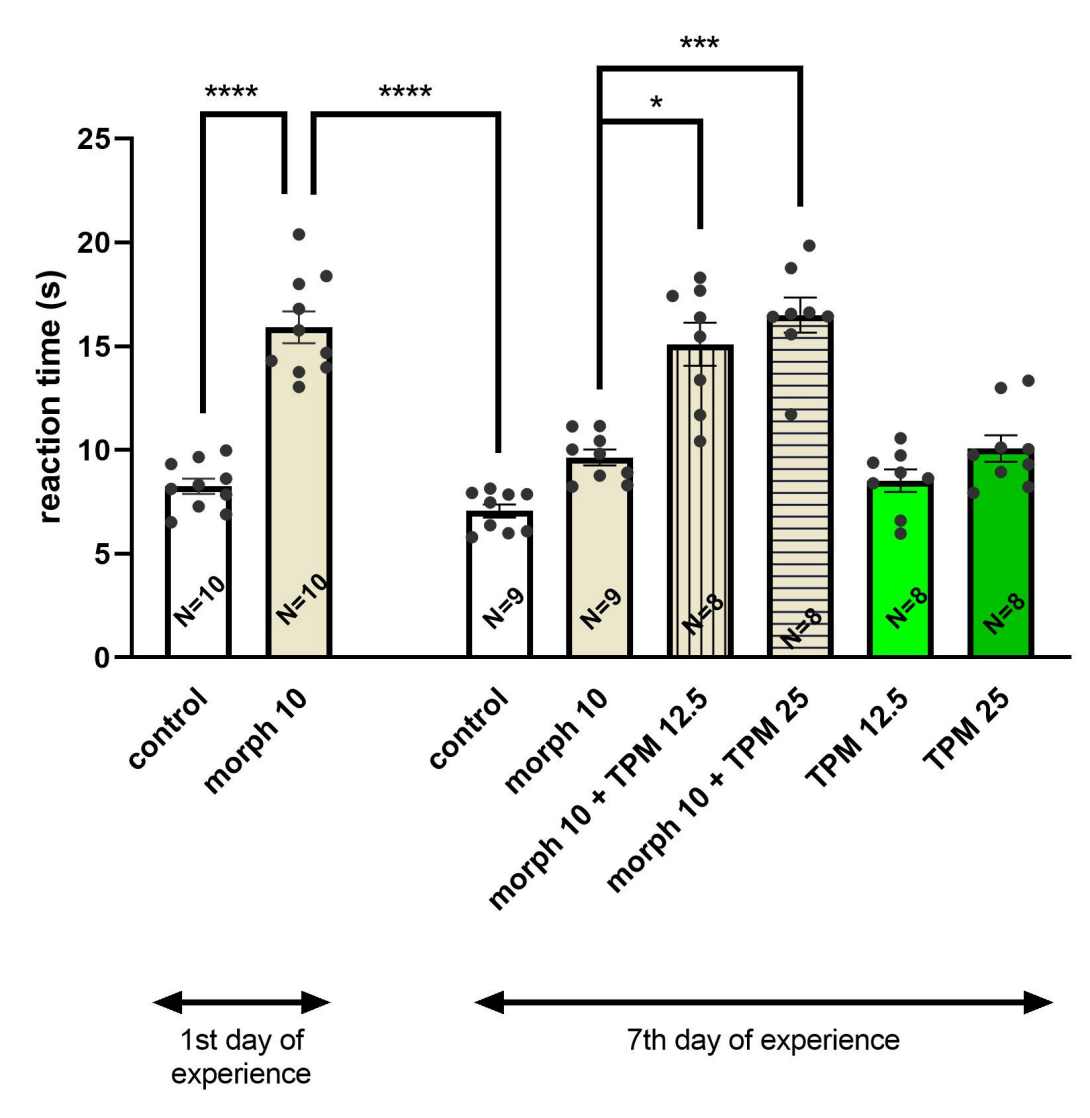
Effect of topiramate (TPM) (12.5; 25 mg/kg, i.p.) on the expression of tolerance to the antinociceptive effect of morphine (morph) (10 mg/kg, i.p.) in the hot-plate test in mice; * *p* < 0.05; *** *p* < 0.001; **** *p* < 0.0001 (Tukey’s test). Total number of mice in this experiment = 70.

In the Tukey’s test, it was confirmed that a single dose of morphine significantly (*p* < 0.0001) increased the response time of the animals as compared to the control group. Chronic morphine administration, however, significantly reduced (*p* < 0.0001) the response time in mice compared to the time measured after a single morphine injection. The administration of topiramate (12.5 and 25 mg/kg) with morphine on the last day of the experiment significantly prolonged the response time of the mice (*p* < 0.05 and *p* < 0.001, respectively) in comparison with the time observed after chronic morphine administration. The administration of topiramate alone at both doses did not influence animal behavior as compared to the control group (Figure 1).

#### 3.1.2. The Influence of Topiramate on the Acquisition of Morphine Tolerance

**Figure 2:**
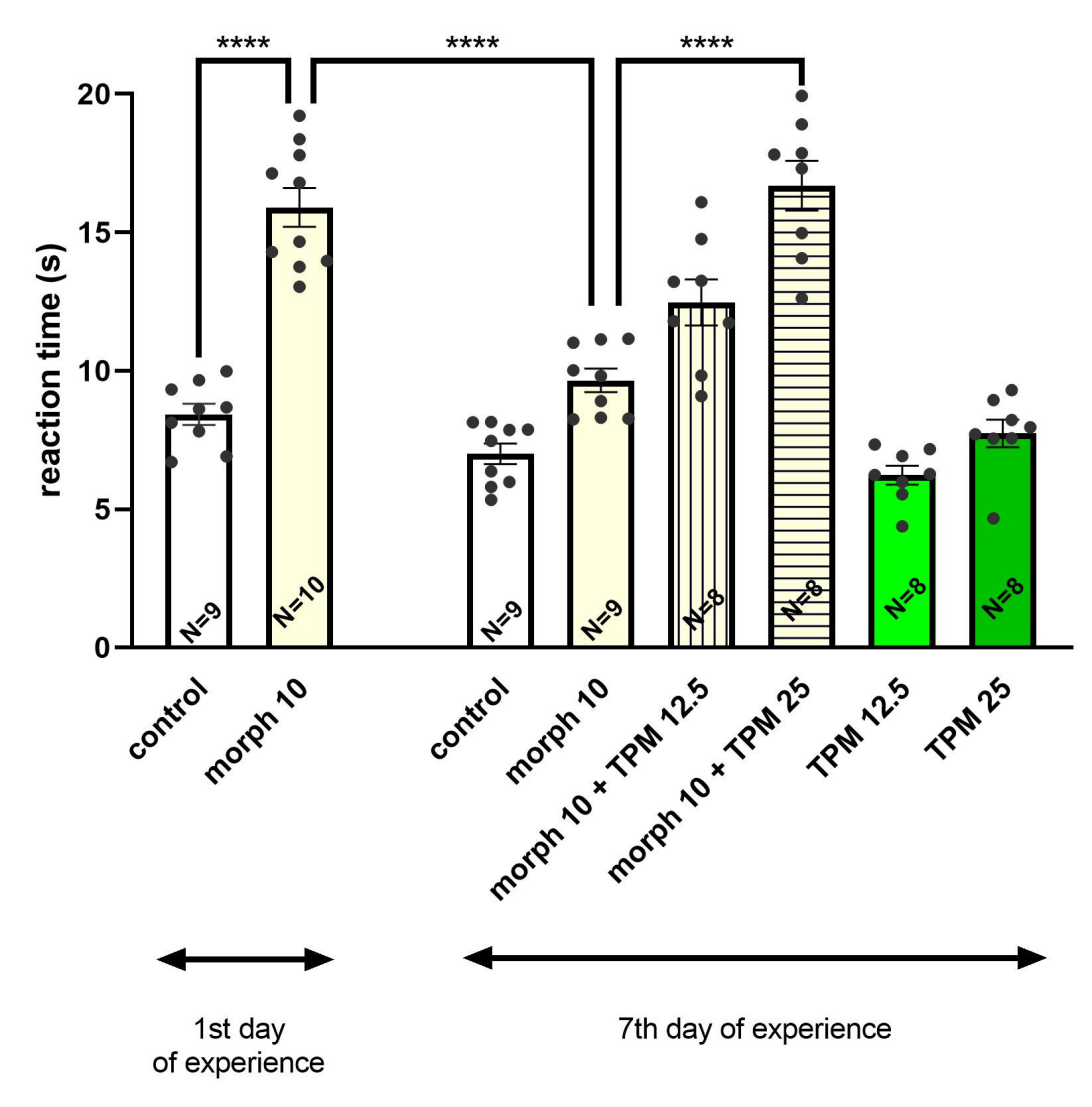
Effect of topiramate (TPM) (12.5; 25 mg/kg, i.p.) on the acquisition of tolerance to the antinociceptive effect of morphine (morph) (10 mg/kg, i.p.) in the hot-plate test in mice; **** *p* < 0.0001 (Tukey’s test). Total number of mice in this experiment = 69.

Significant differences in mice behavior were revealed in the two-way ANOVA following morphine administration on days 1 and 7 of the experiment compared to the control group. A day effect (F(1,32) = 55.68; *p* < 0.0001), drug effect (F(1,32) = 97.13, *p* < 0.0001), and interaction effect (F(1,32) = 22.26, *p* < 0.0001) were observed. Additionally, one-way ANOVA confirmed statistically significant changes in the behavior of mice on day 7 of the experiment (F(5,48) = 22.41; *p* < 0.0001) (Figure 2).

In the Tukey’s test, it was demonstrated that an acute dose of morphine significantly (*p* < 0.0001) increased the response time of mice to the nociceptive stimulus as compared to control animals. The chronic exposure to morphine significantly (*p* < 0.0001) shortened the reaction time of mice in comparison with the effect observed after acute morphine doses. The chronic administration of a higher dose of topiramate (25 mg/kg) along with morphine, significantly (*p* < 0.0001) increased the response time of animals as compared to the effect observed in mice chronically exposed to morphine. The chronic administration of topiramate at both doses had no effect on the behavior of mice in comparison with the control animals (Figure 2).

### 3.2. Effect of Topiramate (12.5; 25 mg/kg, i.p.) on the Severity of Morphine Withdrawal Symptoms Induced by Naloxone (2 mg/kg, i.p.) in Mice

#### 3.2.1. The Influence of Topiramate on the Expression of Morphine Withdrawal Symptoms

Based on one-way ANOVA, it was shown that a single dose of topiramate in morphine-dependent animals resulted in statistically significant differences in the number of jumps (F(6,59) = 13.01; *p* < 0.0001).

**Figure 3:**
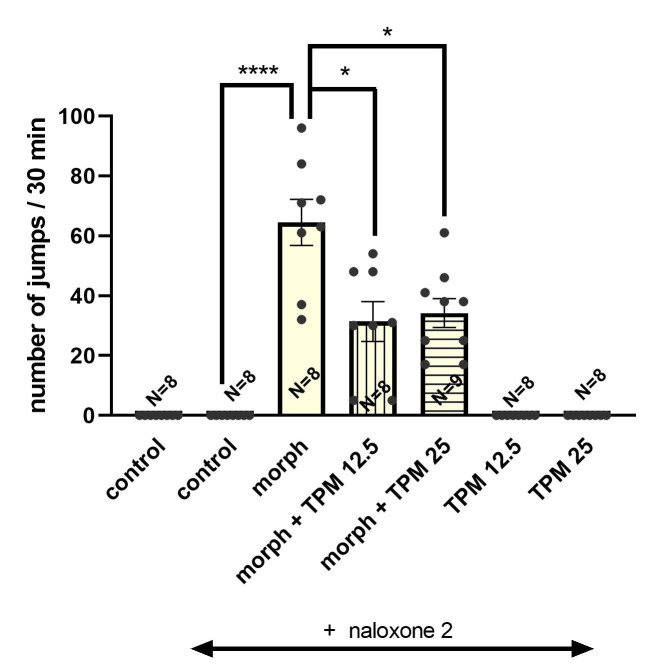
Effect of topiramate (TPM) (12.5; 25 mg/kg, i.p.) on the expression of morphine (Morph) withdrawal symptoms induced by naloxone (2 mg/kg, i.p.) in mice; * *p* < 0.05; **** *p* < 0.0001 (Tukey’s test). Total number of mice in this experiment = 57.

It was confirmed in post hoc test that an acute dose of naloxone given in mice chronically treated with morphine significantly (*p* < 0.0001) increased the number of jumps as compared to animals that did not receive morphine. The administration of acute doses of topiramate (12.5 and 25 mg/kg), significantly (*p* < 0.05) reduced the number of jumps in morphine-dependent mice. Topiramate alone did not influence the behavior of mice which were not treated with morphine (Figure 3).

#### 3.2.2. The Influence of Topiramate on the Acquisition of Morphine Withdrawal Symptoms

**Figure 4:**
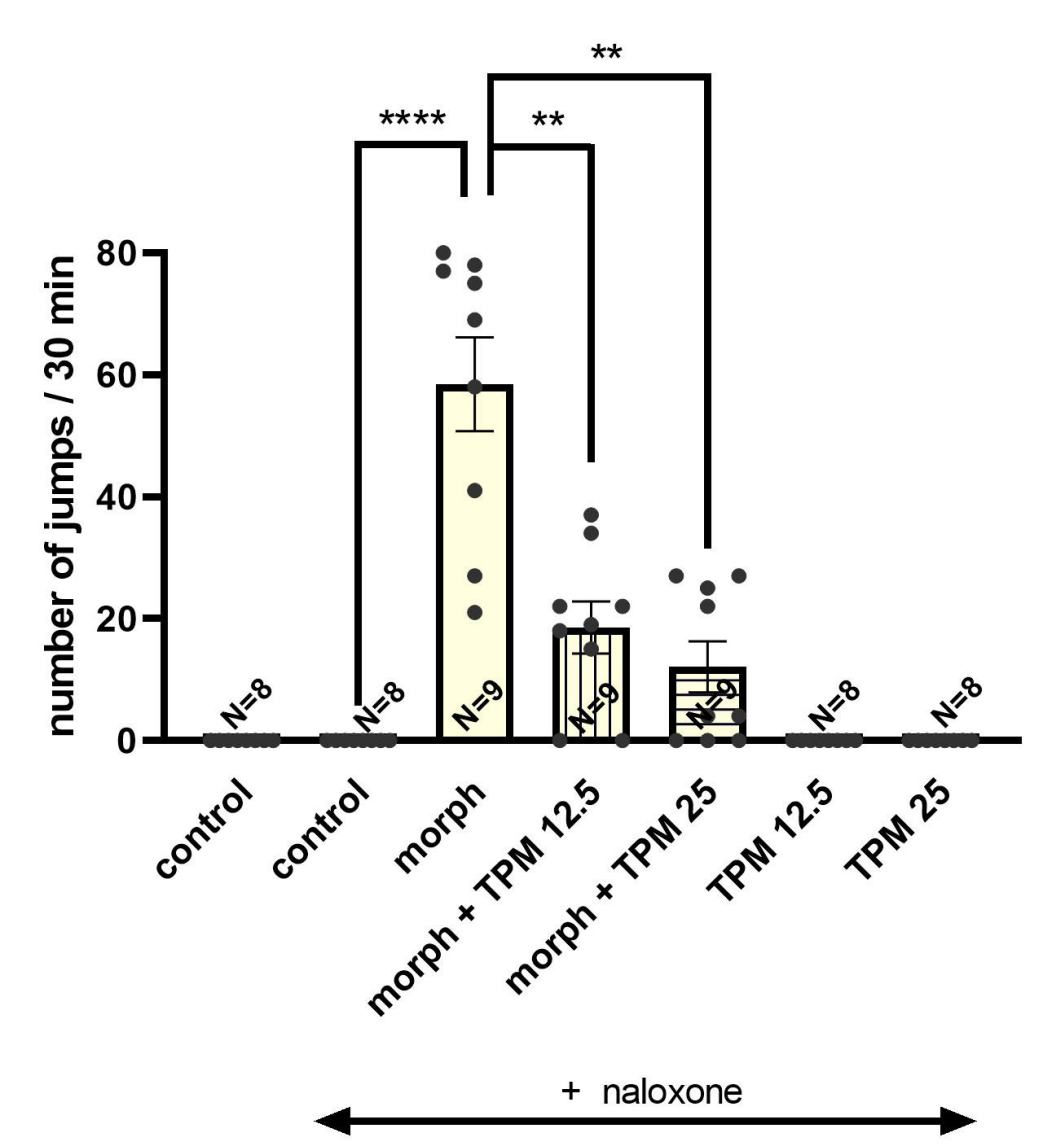
Effect of topiramate (TPM) (12.5; 25 mg/kg, i.p.) on the acquisition of morphine (Morph) withdrawal symptoms Induced by naloxone (2 mg/kg, i.p.) in mice; ** *p* < 0.01; **** *p* < 0.0001 (Tukey’s test). Total number of mice in this experiment = 59.

One-way analysis of variance (ANOVA) revealed statistical differences in the behavior of mice receiving chronic morphine and/or topiramate (F(6,57) = 8.822; *p* < 0.0001). In the Tukey’s test, it was confirmed that naloxone administration in mice chronically treated with morphine significantly increased (*p* < 0.0001) the number of jumps compared to the number recorded in animals not receiving morphine. Chronic administration of topiramate at both doses (12.5 and 25 mg/kg) significantly reduced (*p* < 0.01) the number of jumps in morphine-dependent animals. Chronic administration of both topiramate doses (12.5 and 25 mg/kg) had no effect on the behavior of mice not chronically treated with morphine (Figure 4).

### 3.3. Effect of Topiramate (12.5; 25; 50 mg/kg, i.p.) on Behavioral Sensitization to the Locomotor Effects of Morphine (10 mg/kg, i.p.) in Mice

#### 3.3.1. The Influence of Topiramate on the Expression of Morphine Sensitization

**Figure 5:**
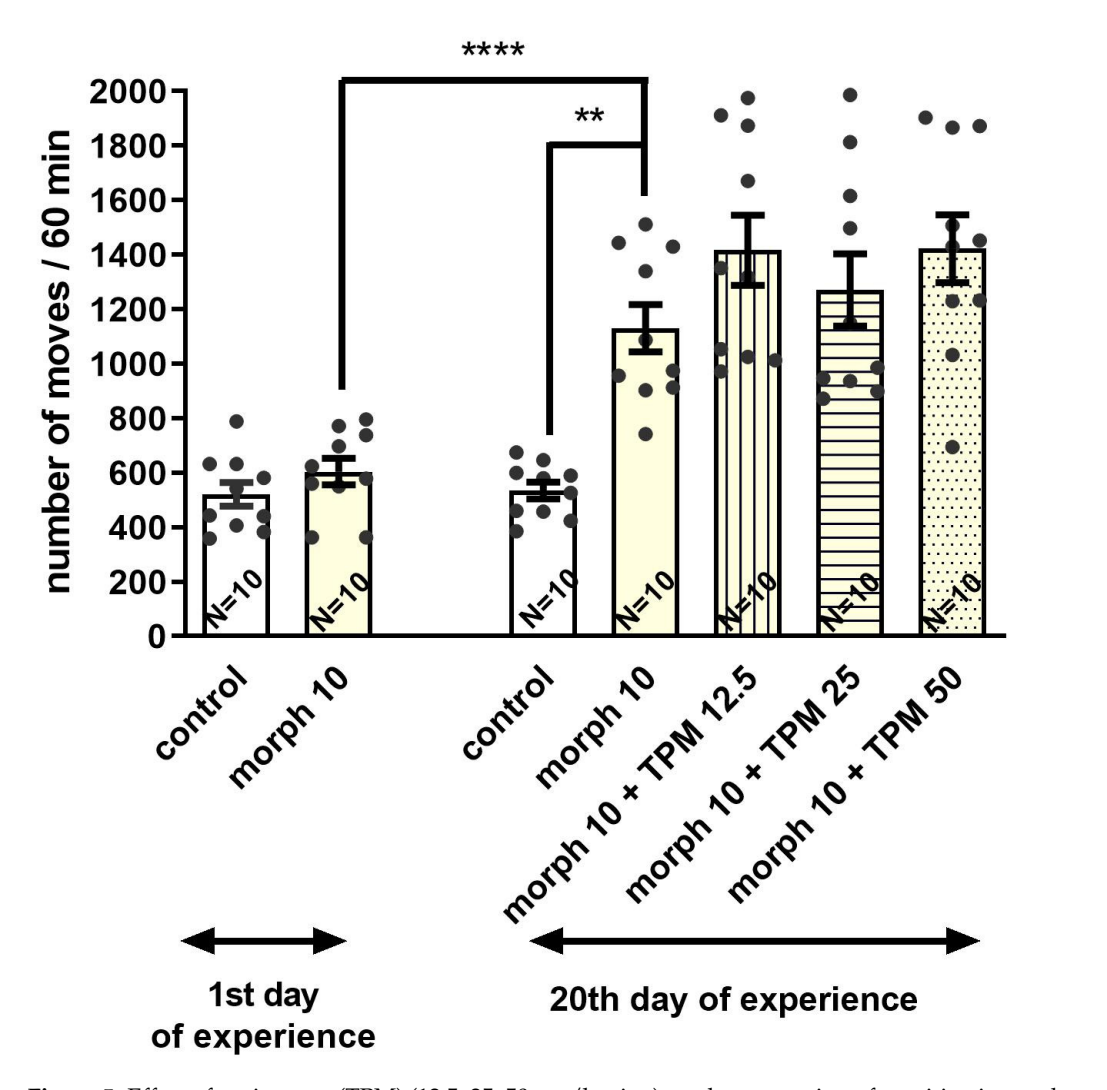
Effect of topiramate (TPM) (12.5; 25; 50 mg/kg, i.p.) on the expression of sensitization to the locomotor effects of morphine (morph) (10 mg/kg, i.p.) in mice; ** *p* < 0.01; **** *p* < 0.0001 (Tukey’s test). Total number of mice in this experiment = 70.

Two-way ANOVA revealed statistical differences in the locomotor activity of animals treated with vehicle and morphine on the 1st and 20th days of the study. A day effect (F(1,32) = 9.079; *p* < 0.0001), drug effect (F(1,32) = 85.45; *p* < 0.0001), and interaction effect (F(1,32) = 9.420; *p* < 0.0001) were observed. In the Tukey’s test, it was shown that an acute dose of topiramate administered on day 20 of the experiment had no effect on the locomotor activity of animals receiving the challenge dose of morphine (10 mg/kg) (Figure 5).

#### 3.3.2. The Influence of Topiramate on the Acquisition of Morphine Sensitization

**Figure 6:**
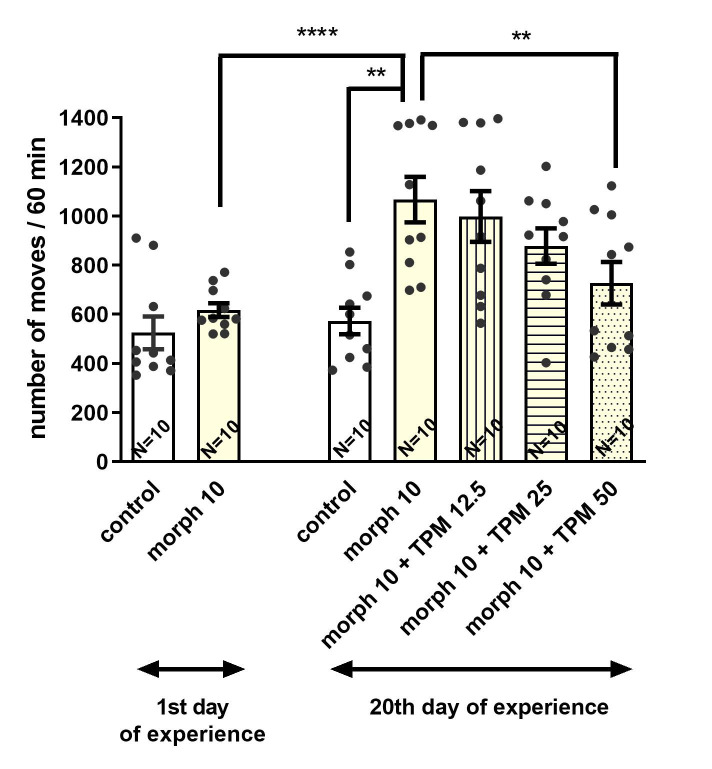
Effect of topiramate (TPM) (12.5; 25; 50 mg/kg, i.p.) on the acquisition of sensitization to the locomotor effects of morphine (Morph) (10 mg/kg, i.p.) in Mice; ** *p* < 0.01; **** *p* < 0.0001 (Tukey’s test). Total number of mice in this experiment = 70.

On the 20th day of the study, the one-way analysis of variance revealed that the administration of the challenge dose of morphine to animals receiving intermittent doses of both morphine and topiramate on days 1, 4, 7, 10, and 13 of the experiment led to significant differences in the locomotor activity of studied animals (F(4,45) = 5.018; *p* = 0.0020). It was confirmed in the post hoc test that the administration of a morphine challenge dose given to animals sporadically treated with morphine significantly increased (*p* < 0.01) the locomotor activity in comparison with the control animals (on day 20 of the experiment). However, sporadic doses of topiramate along with morphine (on days 1, 4, 7, 10, and 13, but not on day 20 of the experiment) significantly reduced (*p* < 0.01) the locomotor activity of animals only when topiramate was administered at the dose of 50 mg/kg compared to the group receiving morphine alone (Figure 6).

## 4. Discussion

**Figure 7:**
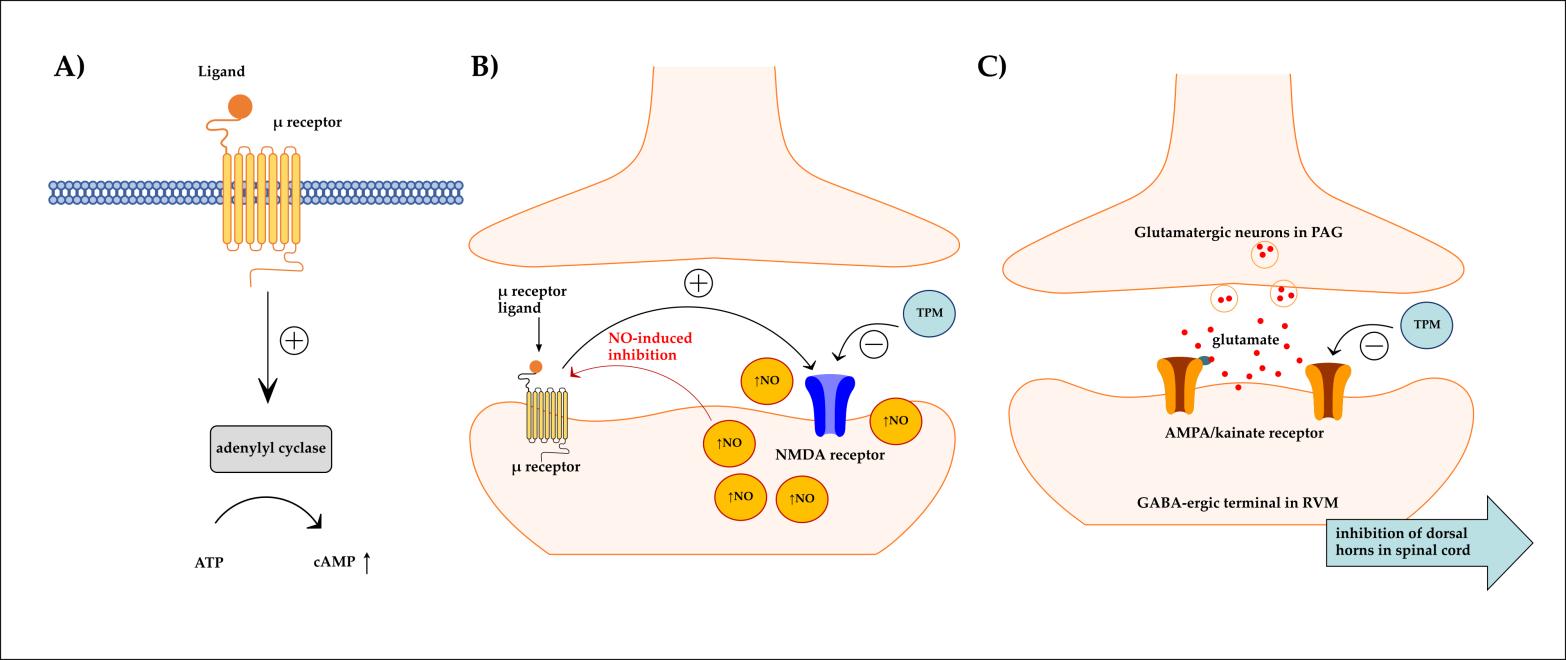
The effect of topiramate on the function of μ-type opioid receptors, NMDA glutamate receptors, AMPA receptors and kainate receptors. Morphine tolerance is an effect of desensitization of μ-type opioid receptors. Desensitization is associated with increased adenylyl cyclase activity and increased cAMP levels in neurons of the locus coeruleus, nucleus accumbens, and others structures (**A**). An important role in desensitization is also attributed to NMDA glutamate receptors in postsynaptic structures. They are co-located with µ receptors in brain areas involved in pain transmission (the dorsal horns of the spinal cord, the nucleus of the locus coeruleus and the periaqueductal gray (PAG)). Stimulation of µ opioid receptors also activates NMDA receptors, and increases the nitric oxide secretion. As a negative feedback, it reduces µ-receptor stimulation (**B**). Additionally, desensitization of opioid receptors may be related to AMPA receptors located in the descending nociceptive pathway (PAG and RVM). PAG sends glutamatergic neurons to the rostroventral medulla (RVM), where they influence on AMPA receptors located on GABAergic terminals. It leads to inhibition of signals in the dorsal horns of the spinal cord (**C**). Topiramate blocks mainly AMPA receptors and kainate receptors. Topiramate blocks also NMDA receptors, but this effect is weaker.

The major achievement of the present study is demonstration that topiramate, a new-generation anticonvulsant, may be an effective drug in reducing some symptoms of morphine dependence in mice, including morphine tolerance, morphine withdrawal signs, and morphine-induced behavioral sensitization. Firstly, topiramate effectively inhibited morphine tolerance, which was evidenced in the hot-plate test in mice. The development of morphine tolerance was achieved by its repeated administration (7 days) at a constant dose (10 mg/kg). On the first and seventh day of morphine administration, the hot-plate test was performed and animal reaction for nociceptive stimulus was compared. We showed that on the seventh day of morphine administration, the animals’ response to the thermal stimulus was significantly shortened, which confirmed the development of tolerance in mice. The administration of topiramate inhibited both the expression and acquisition of morphine tolerance to the antinociceptive effect. An acute exposure of mice to both doses of topiramate produced the inhibition of morphine tolerance. In the case of chronic topiramate exposure, the inhibition of morphine tolerance was observed only after the higher dose of topiramate. It should be emphasized that the effect of topiramate in morphine tolerance has not yet been studied. It is currently believed that the major cause of morphine tolerance is the desensitization of μ-type opioid receptors, which is mainly associated with increased adenylyl cyclase activity and increased cAMP levels in neurons of the locus coeruleus, nucleus accumbens, and others [51]. Moreover, an important role in desensitization is also attributed to NMDA glutamate receptors in postsynaptic structures [52]. They are co-located with µ receptors in brain areas involved in pain transmission (the dorsal horns of the spinal cord, the nucleus of the locus coeruleus, and the periaqueductal gray (PAG)). Furthermore, the stimulation of µ opioid receptors activates NMDA receptors, and increases the nitric oxide secretion. As a negative feedback system, it reduces µ-receptor stimulation [53,54,55]. In addition, the desensitization of opioid receptors may be related to the distribution of AMPA receptors in the descending nociceptive pathway (PAG and RVM) [56]. PAG sends glutamatergic neurons to the rostroventral medulla (RVM), where they influence AMPA receptors located on GABAergic terminals. This leads to the inhibition of signals in the dorsal horns of the spinal cord [56]. Taking into account the fact that the mechanism of topiramate action is associated with inhibition of AMPA receptors and kainate receptors [7,8,9,10], it may be supposed that the attenuation of morphine tolerance by topiramate observed in the present experiment can be attributed to AMPA receptors, Figure 7.

In the second step, the effect of topiramate on the intensity of morphine withdrawal signs was investigated. A typical model of physical dependence on morphine was used, in which withdrawal symptoms were induced by naloxone. It was shown that topiramate significantly reduced the severity of morphine withdrawal symptoms—this effect was observed after the administration of both doses of topiramate and after single and chronic exposure to that compound. Other authors have shown that topiramate reduced the severity of morphine withdrawal symptoms, but it was given acutely at significantly higher dose, i.e., 40 mg/kg, [57]. Moreover, Hajhashemi and Abed-Natanzi [58] observed a similar effect of topiramate after a single exposure at a dose of 100 mg/kg. To date, there are no studies demonstrating the effect of chronic exposure to topiramate on the intensity of morphine withdrawal symptoms.

It is known that the appearance of morphine withdrawal symptoms is an effect of a deep decrease in dopamine concentration in the nucleus accumbens [59]. Other neurotransmitters and neuromodulators, such as glutamate, noradrenaline, or GABA, also take part in this phenomenon [60,61,62]. Although other mechanisms cannot be excluded, it can be assumed that topiramate abolished morphine withdrawal symptoms via increasing GABAergic transmission [13].

Finally, the influence of topiramate on behavioral sensitization to the locomotor effects of morphine in mice was assessed. The behavioral sensitization was achieved by chronic, intermittent administration of an ineffective morphine dose. Then, after a 7-day drug-free period, a challenge dose of morphine was administered. After each morphine injection, in order to associate the morphine injection with the environment, the animals were placed in apparatus used for the measurement of animal mobility. It was shown that the challenge morphine dose evoked a significant increase in the locomotor activity of mice sporadically treated with morphine, confirming the development of behavioral sensitization. Topiramate did not produce any spectacular effect—a single dose of topiramate had no effect on morphine behavioral sensitization, while chronic exposure to that compound significantly inhibited morphine sensitization only when the highest dose of topiramate was administered.

Thereby, it was confirmed that topiramate has a limited effect on attenuation of morphine-seeking behavior in mice. It has been shown that the expression of sensitization is primarily associated with an increase in dopamine secretion in mesolimbic brain areas [63,64] and is frequently manifested as an increase in the locomotor activity of animals. Later studies have demonstrated that adaptive changes in the mesolimbic system occur not only within dopaminergic transmission but also in glutamatergic transmission [65,66,67,68] with both of NMDA [69] and AMPA receptors [70]. It seems that the weaker effect of topiramate in morphine sensitization in the present study may result from its mechanism of action, i.e., a stronger effect on the activity of AMPA receptors than NMDA receptors. Until now, there have been no scientific reports on the role of topiramate in morphine-induced behavioral sensitization. Thus, the present study points to a new direction which seems to be important in the suppression of cravings and relapses into drug use.

The limitation of this study is that we performed only behavioral experiments, but not molecular experiments. Thus, based on the obtained findings, it is impossible to define a new clinical use for topiramate; rather, this study offers a novel perspective for the therapy of morphine dependence. Thus, more results are needed to fully recognize the significance of topiramate in the attenuation of morphine dependence.

## 5. Conclusions

In summary, in the present study, the beneficial role of topiramate in morphine dependence was shown in mice. Although this achievement requires more studies, it seems that topiramate can be considered as a potential drug in the management of morphine dependence in people. The present study extend the current knowledge on the pharmacological activities of topiramate and may be useful in searching for new ideas in the pharmacotherapy of morphine dependence.

## Data Availability

The original contributions presented in this study are included in the article. Further inquiries can be directed to the corresponding authors.

## Abbreviations

The following abbreviations are used in this manuscript:

i.p.	intraperitoneal injection
GABA	gamma-aminobutyric acid
NMDA	N-methyl-D-aspartate
AMPA	aminomethylphosphonic acid
RVM	rostroventral medulla
N	the number of animals per group
PAG	periaqueductal gray
Morph	morphine
p	probability ratio
TPM	topiramate
